# Escalating drought sensitivity of a greening China

**DOI:** 10.3389/fpls.2026.1873724

**Published:** 2026-08-05

**Authors:** Yanan Cao, Zhipeng Wang, Ben Niu, Xutong Li, Fei Liu, Mengtian Lu, Yunpu Zheng, Xianzhou Zhang

**Affiliations:** 1 School of Earth Science and Engineering, Hebei University of Engineering, Handan, China; 2 Hebei Key Laboratory of Intelligent Water Conservancy, School of Water Conservancy and Hydropower, Hebei University of Engineering, Handan, China; 3 Key Laboratory of Ecosystem Network Observation and Modeling, Institute of Geographic Sciences and Natural Resources Research, Chinese Academy of Sciences, Beijing, China; 4 College of Urban Construction, Nanjing Tech University, Nanjing, China

**Keywords:** China, drought sensitivity, ecosystem adaptation, multi-timescale SPEI, vegetation index

## Abstract

**Introduction:**

The sensitivity of vegetation to drought is a critical determinant of whether recurrent droughts will disrupt sustainable greening and climate change mitigation efforts. However, its potential spatiotemporal heterogeneity, shaped by both environmental and anthropogenic drivers, remains poorly understood.

**Methods:**

Here, we employed multiscalar Standardized Precipitation Evapotranspiration Index (SPEI) to unfold the spatiotemporal patterns of drought across China since the 1980s. We then examined the sensitivity of land surface greenness to drought and its changes across drainage basins, agricultural regions, and plant functional types.

**Results:**

China has experienced a notable aridification trend since the 1980s, primarily driven by intensified spring dryness across the northern drylands. Significant correlations between greenness variability and SPEI were observed across 29.09% to 44.37% of vegetated lands, with the spatial extent increasing along with the SPEI timescale (12 to 48 months). Critically, despite a widespread greening trend covering nearly 55% of vegetated lands, the sensitivity of greenness variability to multiscalar SPEI markedly escalated across 27.48%–39.67% of China.

**Discussion:**

Our findings suggest that increasing drought sensitivity poses a growing potential constraint to the sustainability of vegetation greening in China, highlighting the urgent need for adaptive water resource management and ecosystem restoration strategies under a warming climate.

## 1 Introduction

Drought is a prevalent extreme climatic event that is likely to increase in scope and frequency due to climate warming, imposing water stress on vegetation growth and threatening regional and global surface greening (Lesk et al., 2016; Pokhrel et al., 2021; Santini et al., 2022; Vicente-Serrano et al., 2020). Generally, the severity of drought to an ecosystem depends on not only the drought regime (e.g., frequency, duration and magnitude) but also the ecosystem’s sensitivity, which reflects the magnitude of vegetation response to water deficits. This sensitivity is determined by community composition, physiological adaptations, and environmental modulators that either directly alleviate water stress or indirectly compensate for growth reductions (Chen et al., 2022a; Li et al., 2023a; Tan et al., 2021). Consequently, drought sensitivity is not static but varies across space and time under global change (Jiao et al., 2021; Li et al., 2024; Sloat et al., 2018; Zeng et al., 2022). However, how this sensitivity has shifted amid widespread global greening, and the associated risks to ecosystem stability, remain insufficiently evaluated.

Over the past four decades, the Earth’s terrestrial surface has experienced a substantial greening trend, characterized by significant increases in leaf area index (LAI) and vegetation greenness (Chen et al., 2019; Huang et al., 2017; Myers-Smith et al., 2020; Zhu et al., 2016). China has emerged as a major contributor to this global greening, accounting for approximately 25% of the global net increase in vegetation area despite comprising only 6.6% of global vegetated land (Chen et al., 2019). This remarkable greening achievement has been primarily driven by large-scale ecological restoration programs, including the Three-North Shelter Forest Program, the Grain for Green Project, and the Natural Forest Protection Program (Piao et al., 2019; Yue et al., 2024). Land-use management changes have contributed approximately 72.7% to China’s terrestrial carbon sink, with afforestation and reforestation accounting for 49.0% (Yue et al., 2024). While these efforts have substantially enhanced vegetation coverage and ecosystem productivity, questions remain regarding the long-term sustainability of such greening trends under intensifying drought conditions. Concurrent with widespread greening, many regions of the world, particularly arid and semi-arid areas, have experienced significant drying trends. Since the 1980s, global drought frequency, duration, and severity have increased markedly under a warming climate (Pokhrel et al., 2021). This change poses a critical challenge, whether the expansion of vegetation cover increases water demand, thereby exacerbating water stress and making ecosystems more vulnerable to drought events. A recent study has revealed that vegetation greening has accelerated hydrological drought in approximately two-thirds of China’s river basins, with drought-affected areas increasing by 2–9% and drought duration extending by 0.035–0.131 months (Lan et al., 2024). This “greening but drying” paradox highlights the urgent need to understand how vegetation sensitivity to drought is evolving in the context of ecological restoration.

The sensitivity of vegetation to drought is inherently complex and varies across multiple dimensions. Recent advances have demonstrated that drought sensitivity exhibits substantial spatial heterogeneity, with different biomes responding differently to water deficits depending on climate zones, vegetation types, and soil characteristics. For instance, Zhang et al. (2022b) suggested that the arid regions experienced precipitation sensitivity upward at a rate of 0.624% yr^-^¹ over the past four decades, while humid regions have shown decreasing sensitivity at −0.618% yr^-^¹. They conclude that the divergence is primarily driven by the contrasting effects of CO_2_ fertilization. The elevated atmospheric CO_2_ reduces stomatal conductance (conserving water at the leaf level) but simultaneously promotes leaf area expansion, which can increase total canopy water demand, particularly in water-limited lands (Zhang et al., 2022b). Liu et al. (2024) provided evidence that climate-driven disturbances (e.g., drought and fire) can amplify the sensitivity for forest gross primary productivity (GPP) to drought, with approximately 30% of affected areas never recovering to pre-disturbance sensitivity levels. Other factors, such as irrigation and fertilization in farmland management, may reduce the sensitivity of photosynthetic productivity to drought by alleviating water stress or compensating for photosynthetic losses (Li et al., 2023a; Wang et al., 2025; Zhao et al., 2023). Such complex interactions create significant uncertainties in predicting ecosystem responses to future climate change.

Furthermore, the temporal dimension of drought-vegetation relationships has received increasing attention. The development of multiscalar drought indices, particularly the Standardized Precipitation Evapotranspiration Index (SPEI), has enabled researchers to examine vegetation responses across different drought timescales (Vicente-Serrano et al., 2010, 2013). Different vegetation types exhibit varying sensitivities to drought at different timescales. Generally, short-term droughts (less than 3 months) primarily affect shallow-rooted vegetation through rapid soil moisture depletion, while long-term droughts (more than 12 months) impact deep-rooted forests through groundwater depletion and legacy effects (Anderegg et al., 2015; Vicente-Serrano et al., 2013). Despite these advances, critical knowledge gaps persist regarding the spatiotemporal dynamics of drought sensitivity under widespread greening. While numerous studies have examined static sensitivity patterns, the temporal evolution of sensitivity remains poorly understood, particularly whether vegetation is becoming more or less sensitive to drought over time.

In this study, we intend to employ the multiscalar SPEI to unfold the spatiotemporal patterns of aridity across China since the 1980s. We then try to examine the sensitivity of land surface greenness to drought and its changes across multiple spatial dimensions, including drainage basins, agricultural regions, and plant functional types. Specifically, we addressed the following questions: (1) How has aridity evolved spatially and temporally across China over the past four decades? (2) What is the spatial pattern of vegetation sensitivity to drought at different timescales? (3) Has this sensitivity changed over time, and what are the spatial patterns of such changes? We aim to provide critical insights into the sustainability of vegetation greening under climate change and inform adaptive ecosystem management strategies.

## 2 Materials and methods

### 2.1 Study area

**Figure 1:**
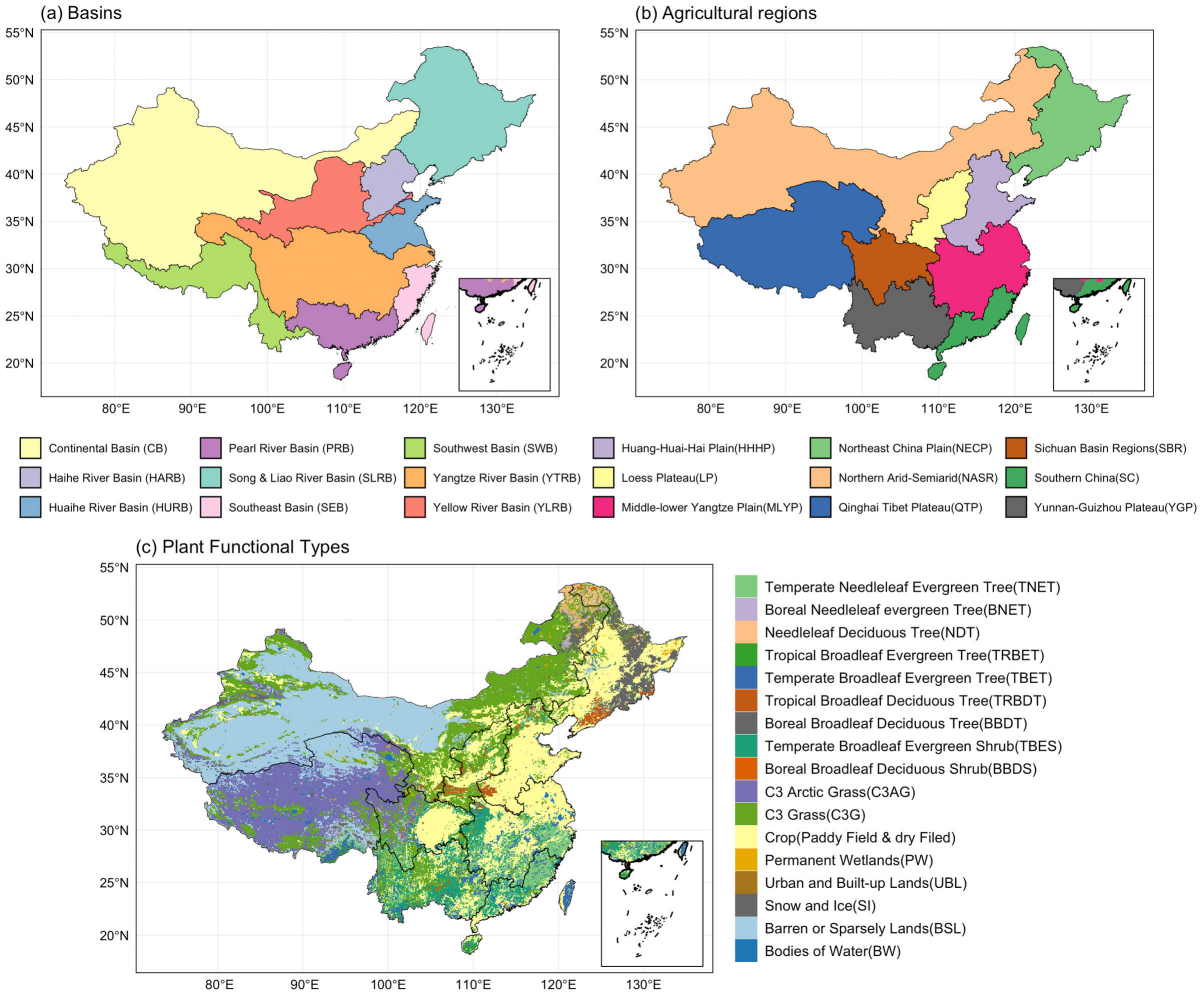
Spatial boundaries of the river basins **(a)**, agricultural regions **(b)**, and spatial distribution of Plant Functional Types **(c)** across China.

Located in East Asia, China has a very diverse landscape with complex mountains and a strong climate gradient. To better understand the different ecohydrological responses, we divided our study area using three layers: major river basins, agricultural regions, and plant functional types (PFTs) (**Figure 1**). The climate changes from a very dry environment in the northwest, mostly made up of the arid Continental Basins (CB) and the dry Loess Plateau (LP), to a wet environment in the southeast, which includes the Pearl River Basin (PRB) and the Middle-lower Yangtze Plain (MLYP). This spatial difference leads to clear regional water shortages. In addition, vegetation changes within these basins are strongly affected by land-use choices and human activities. In the dry northern areas (such as the Loess Plateau and the Northern Arid-Semiarid Region), large ecological restoration projects like the “Grain for Green” program have greatly increased leaf area and forest cover (Fu et al., 2017). This has deeply changed local water-use habits and how plants react to drought (Chen et al., 2025). These managed areas, along with natural ecosystems, ranging from the high alpine grasslands of the Qinghai-Tibet Plateau (QTP) to the tropical forests in Southern China (SC), provide a great area to study how water supply and plant growth connect under long-term environmental change.

### 2.2 Data sources

#### 2.2.1 Multiscalar SPEI dataset

Hiring the Standardized Precipitation Evapotranspiration Index (SPEI) to characterize dryness, we obtained monthly gridded SPEI with multiple timescales from the Global SPEI database (SPEIbase v2.9.0), including 3-month (SPEI-03), 12-month (SPEI-12), 24-month (SPEI-24), 36-month (SPEI-36), and 48-month (SPEI-48). Based on the formula proposed by Vicente-Serrano et al. (2010), these globally long-term SPEI records were produced using monthly precipitation data provided by the Climatic Research Unit (CRU TS 4.07) and potential evapotranspiration (PET) data estimated using the FAO-56 Penman-Monteith method (https://spei.csic.es/) (Beguería et al., 2010, 2014). The SPEI has a spatial resolution of 0.5°×0.5° and covers the whole terrestrial land. In this study, we selected the timeframe spanning from 1982 to 2022.

#### 2.2.2 Land greenness indicator and plant functional classification

The Normalized Difference Vegetation Index (NDVI) is widely employed to assess the dynamics of land surface greenness and plant growth. In this study, we adopted a new version of NDVI data, termed PKU GIMMS NDVI (Version 1.2), which covers the period from 1982 to 2022 and provides biweekly global NDVI data with a spatial resolution of 1/12°. The data are produced by a machine learning model that employs 3.6 million Landsat high-quality NDVI samples across the globe, and a data consolidation method has been used for data consolidation based on previous AVHRR and Moderate-Resolution Imaging Spectroradiometer (MODIS) records. It has proven to be a more reliable basis for global research (Li et al., 2023b) (https://essd.copernicus.org/articles/15/4181/2023/essd-15-4181-2023-assets.html). Here, the annual NDVI (hereafter NDVI) was calculated by averaging the biweekly values for each year, and the inter-annual variability of NDVI was retrieved by removing the long-term linear trend of the annual NDVI time series. The plant functional types (PFTs) data over China were obtained from the National Tibetan Plateau Data Center (TPDC), specifically utilizing the “Plant functional types map in China (1 km)” dataset (Ran et al., 2012). This dataset provides a high-resolution (1 km) spatial distribution of major vegetation functional types across China, which served as the baseline for evaluating eco-geographical gradients and PFT-specific vegetation responses to multi-timescale droughts.

To harmonize the spatial mismatch between the 1/12° NDVI data and the 0.5° SPEI data, the NDVI layers were first spatially aggregated using a 6-fold block-averaging factor to aggregate the mean greenness value for each 0.5° grid. Subsequently, nearest-neighbor resampling was applied to ensure strict pixel-by-pixel spatial alignment and boundary matching between the NDVI and SPEI datasets prior to statistical analyses. When the 1 km PFTs layer was spatially harmonized with the NDVI and SPEI datasets, to maintain a high precision, we employed the modal (majority) aggregation method to upscale the 1 km pixels to the spatial resolution of NDVI because the PFTs map contains categorical (non-continuous) land cover classes. Subsequently, the SPEI data were aligned with the NDVI and aggregated PFTs grids using nearest-neighbor resampling, ensuring strict pixel-to-pixel spatial matching across all analytical layers. In order to avoid abrupt changes in the long-term trend, the detrending was performed as a segmented detrend, with the breakpoint year set at 2000. This procedure was achieved through the “detrend” function embedded in the “pracma” packages under the R platform.

### 2.3 Statistical methods

The Sen’s Slope (Sen, 1968) was used to detect the trend of the target variables. The nonparametric Mann-Kendall (M-K) test (Mann, 1945) was applied to test the significance of the trend. These approaches were embedded in the ‘trend’ package and implemented with R4.4.1. The Pearson correlation coefficient (R) between SPEI and the inter-annual variations of the NDVI was used to indicate the sensitivity of vegetation growth to drought (Jiao et al., 2021). A higher positive R between SPEI and NDVI suggests that vegetation growth is more sensitive to drought, while a smaller or even negative value indicates that vegetation growth is less sensitive to drought. A dynamic sensitivity analysis was conducted to examine the dynamics of the NDVI drought sensitivity, which followed previous studies calculating R through a 9-year sliding window (Chen et al., 2025; Wang et al., 2024). Specifically, from 1982 to 2022, there were 33 continuous windows, yielding a time series of R with 33 values at each grid-cell location. Then, trend analysis was applied to detect the long-term trend of drought sensitivity (R).

It should be clarified that the Pearson correlation coefficient was selected as our primary metric because we focus on tracking the temporal synchronicity and relative coupling intensity of ecohydrological co-variations, rather than constructing absolute quantitative predictive equations. Unlike regression-based sensitivity slopes, which are heavily confounded by regional baseline greenness and local plant traits, the dimensionless nature of R filters out background scale anomalies to enable rigorous cross-ecosystem comparisons. Furthermore, compared to other non-linear model (e.g., machine learning), this empirical, parameter-free approach functions transparently across shifting moving-windows without introducing hyperparameter-tuning artifacts. Given that the multi-scalar SPEI is mathematically transformed into a standardized normal distribution, a first-order linear approximation is statistically appropriate and sufficient to isolate the dominant directional co-variation within our tight 9-year temporal windows.

## 3 Results

### 3.1 The spatiotemporal trend of multiscalar SPEI across China

#### 3.1.1 The seasonal dryness change

**Figure 2:**
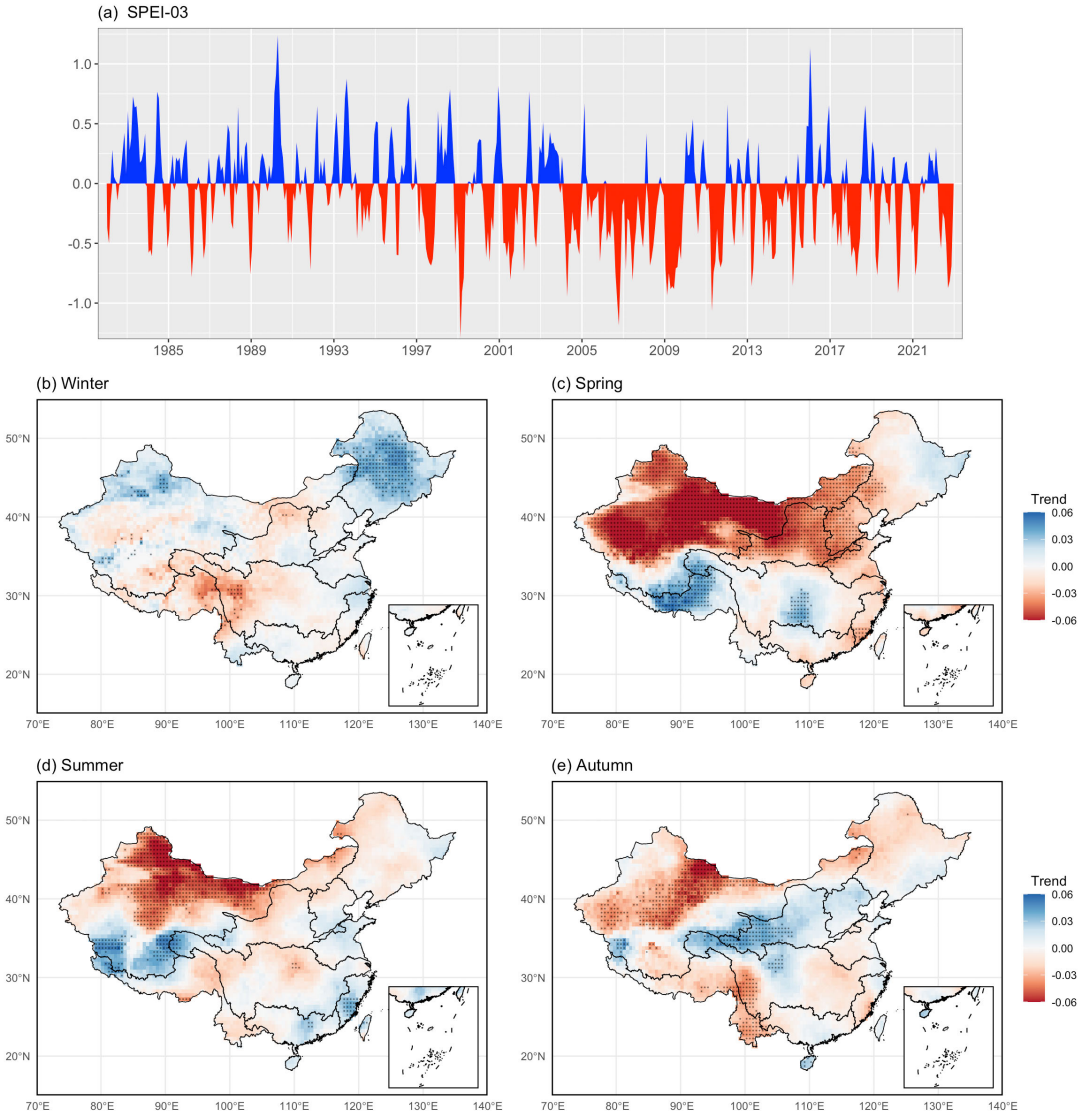
Spatiotemporal patterns of variations in seasonal SPEI. **(a)** shows the temporal variations of SPEI at a 3-month timescale (SPEI-03) overall in China from 1982 to 2022. **(b–e)** present spatial trend (Sen’s Slope) patterns of SPEI in winter (December to February), spring (March to May), summer (June to August), and autumn (September to November), respectively. Land labeled with black dots in **(b–e)** indicates that the trend is statistically significant (M-K test: *p* < 0.05).

Overall, China exhibited an aridification trend during 1982–2022. Since nearly 2000, the frequency of negative SPEI-03 has substantially increased (**Figure 2a**). Specifically, three moderate drought events occurred in February 1999, October 2006, and April 2011 (**Figure 2a**). Spatially, a significantly (*p* < 0.05) wetting trend in winter was mainly observed in the northeast, covering 6.42% of China (**Figure 2b**). In the northern drylands and North China, spring SPEI exhibited a remarkably (*p* < 0.05) negative trend, showing enhanced spring dryness and covering 38.29% of the total area (**Figure 2c**). Conversely, 5.16% of the lands, mainly observed in the southwest Tibetan Plateau, experienced a significant increase in humidity during spring (**Figure 2c**). The proportion of areas with a significant increase in summer dryness was 13.48%, primarily concentrated in the northwest, whereas only 3.31% of lands exhibited a notably wetting trend in the western China (**Figure 2d**). The autumn dryness intensified in the northwest and southwest, accounting for 11.63% of the area (**Figure 2e**). Regions with a significant wetting trend in autumn were predominantly located in the central region, accounting for nearly 4.0% of lands in China (**Figure 2e**).

#### 3.1.2 The annual and multi-yearly dryness change

**Figure 3:**
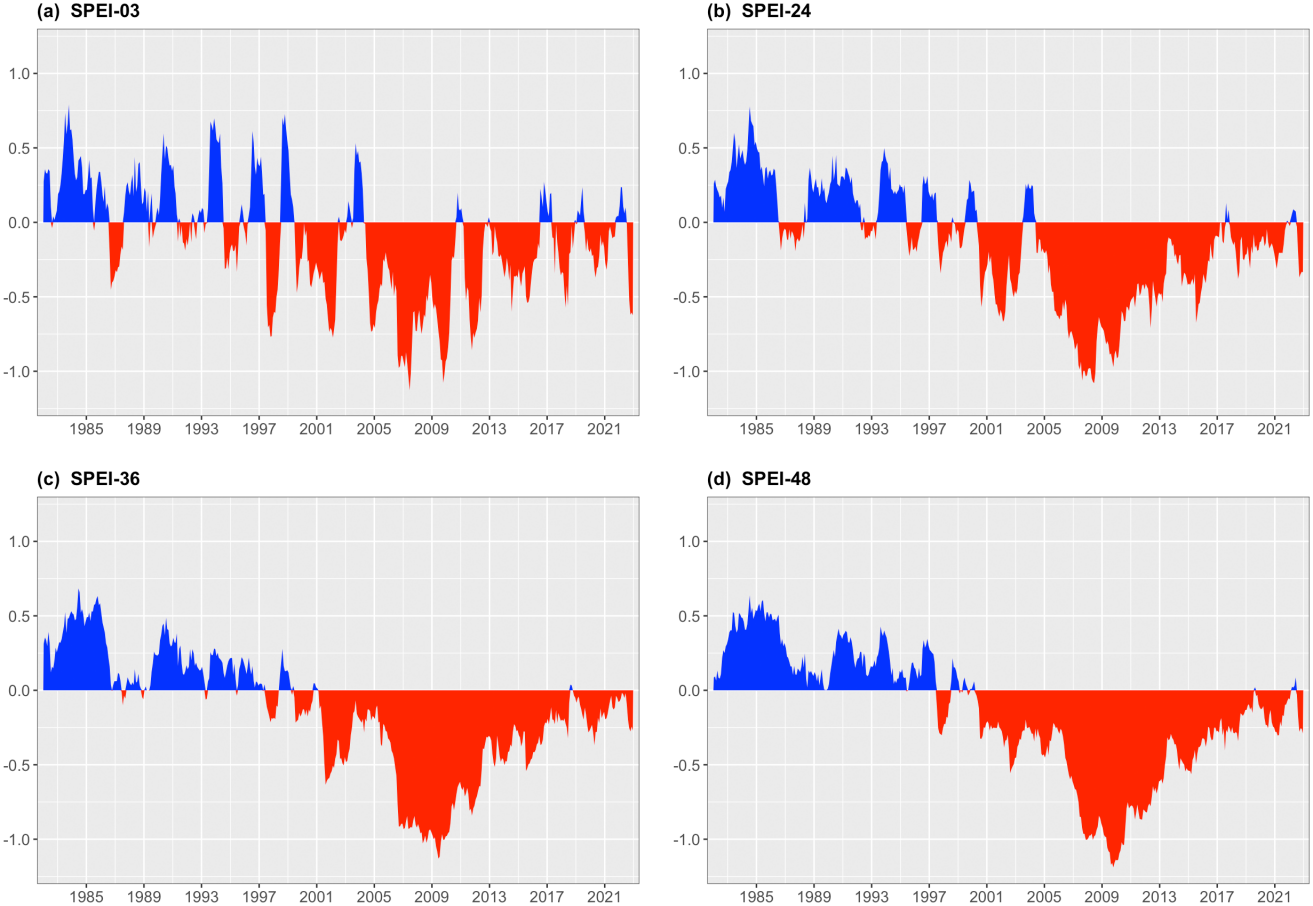
The temporal variations of monthly SPEI-12 **(a)**, SPEI-24 **(b)**, SPEI-36 **(c)**, and SPEI-48 **(d)** across China during 1982–2022.

Since nearly 2000, SPEI at 12-month, 24-month, 36-month, and 48-month timescales across China gradually shifted from a positive phase to a negative phase, suggesting that China entered a relatively dry period (**Figure 3**). Specifically, the dryness reached the peak in the period of 2007–2010, then subsequently decreased in recent years, which was particularly evident in monthly SPEI at multi-year timescales (**Figure 3**).

**Figure 4:**
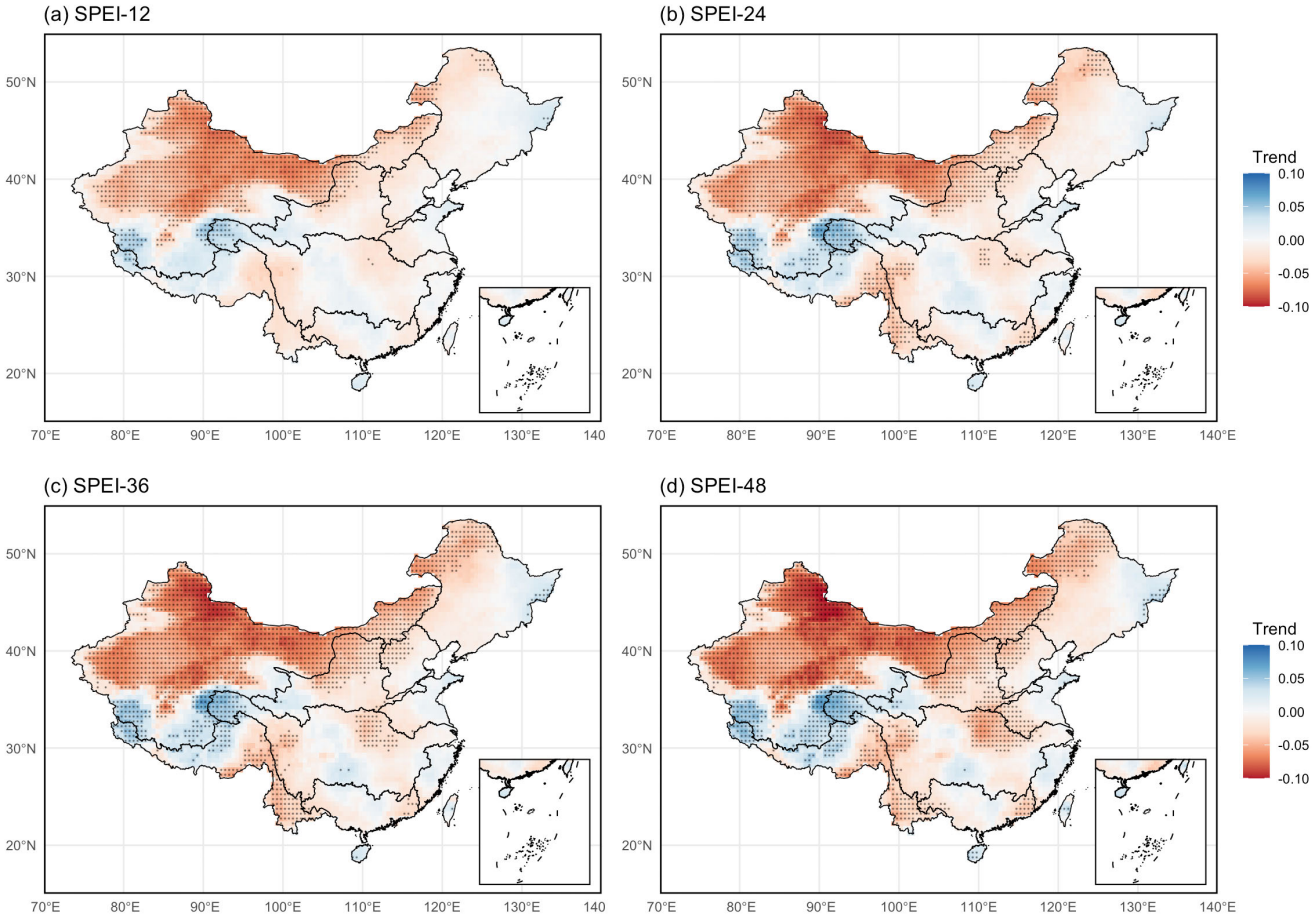
The spatial patterns of yearly and multi-year SPEI trends in China. **(a–d)** respectively denote the spatial trend of SPEI with 12-month (SPEI-12), 24-month (SPEI-24), 36-month (SPEI-36) and 48-month (SPEI-48) time-scale.

Spatially, the multiscalar SPEI, namely SPEI-12, SPEI-24, SPEI-36, and SPEI-48, exhibited a similar change pattern, where a notable decrease trend was found in northwestern China and accounted for 24.05%, 32.50%, 38.47%, and 44.78% of vegetated lands, respectively (**Figure 4**). Specifically, the Continental Basin, widely known as arid and semi-arid regions in China, showed a widely and remarkably (*p* < 0.05) increase in dryness (**Figure 4**). Besides, the downstream of Southwest Basin experienced a significant (*p* < 0.05) drying trend for SPEI-24, SPEI-36, and SPEI-48 (**Figures 4b–d**). The lands showing an obvious humidification trend were very limited, mainly distributed in the Tibetan Plateau in southwest China (**Figure 4**).

### 3.2 The spatiotemporal trend of greenness in China

**Figure 5:**
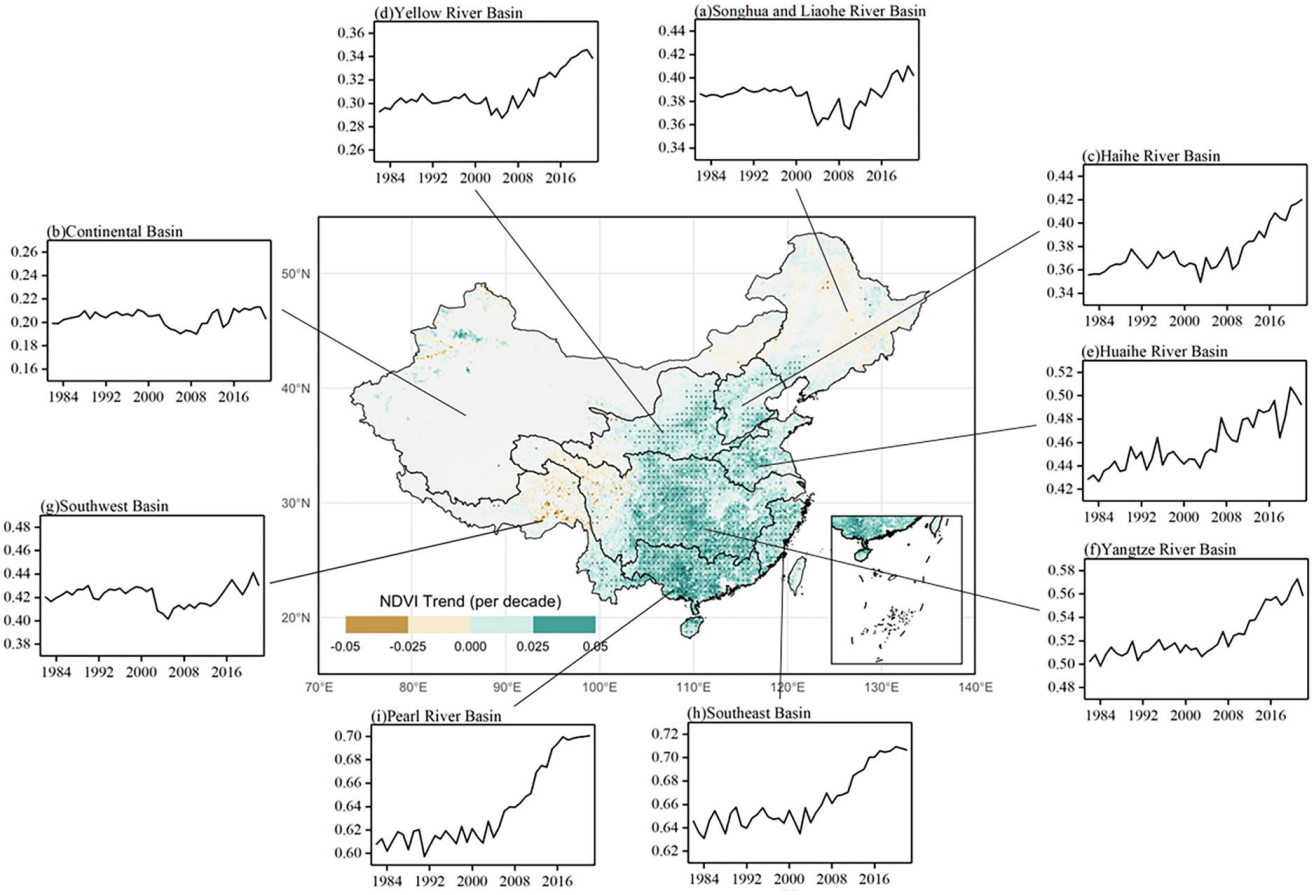
The spatiotemporal trend of annual NDVI across China during 1982–2022. The central panel shows the spatial pattern of NDVI trend (Sen’s slope, ×10^−2^/decade), and the areas labeled with black dots indicate that the trend is statistically significant (M-K test: *p* < 0.05). The inset **(a–i)** indicate NDVI variations in the nine basins in China.

Across more than half (nearly 55%) of vegetated lands in China, NDVI showed a significant increasing trend in the past four decades (**Figure 5**). Spatially, NDVI in the Continental Basin, the Southwest Basin, and the Songhua and Liaohe River Basin, showed no significant change trend (**Figures 5a, b, g**). All other basins, including the Haihe River Basin, the Huaihe River Basin, the Yellow River Basin, the Yangtze River Basin, the Pearl River Basin, and the Southeast Basin, experienced a substantial increase in NDVI, and a higher increase rate was widely observed since the 2000s (**Figures 5c–f, h, i**). Less than 20% of the lands showed a decreasing NDVI, which occurred primarily in parts of the Songhua and Liaohe River Basin and the Southwest Basin (**Figure 5**).

### 3.3 Sensitivity of greenness to SPEI and its trend at pixel scale

**Figure 6:**
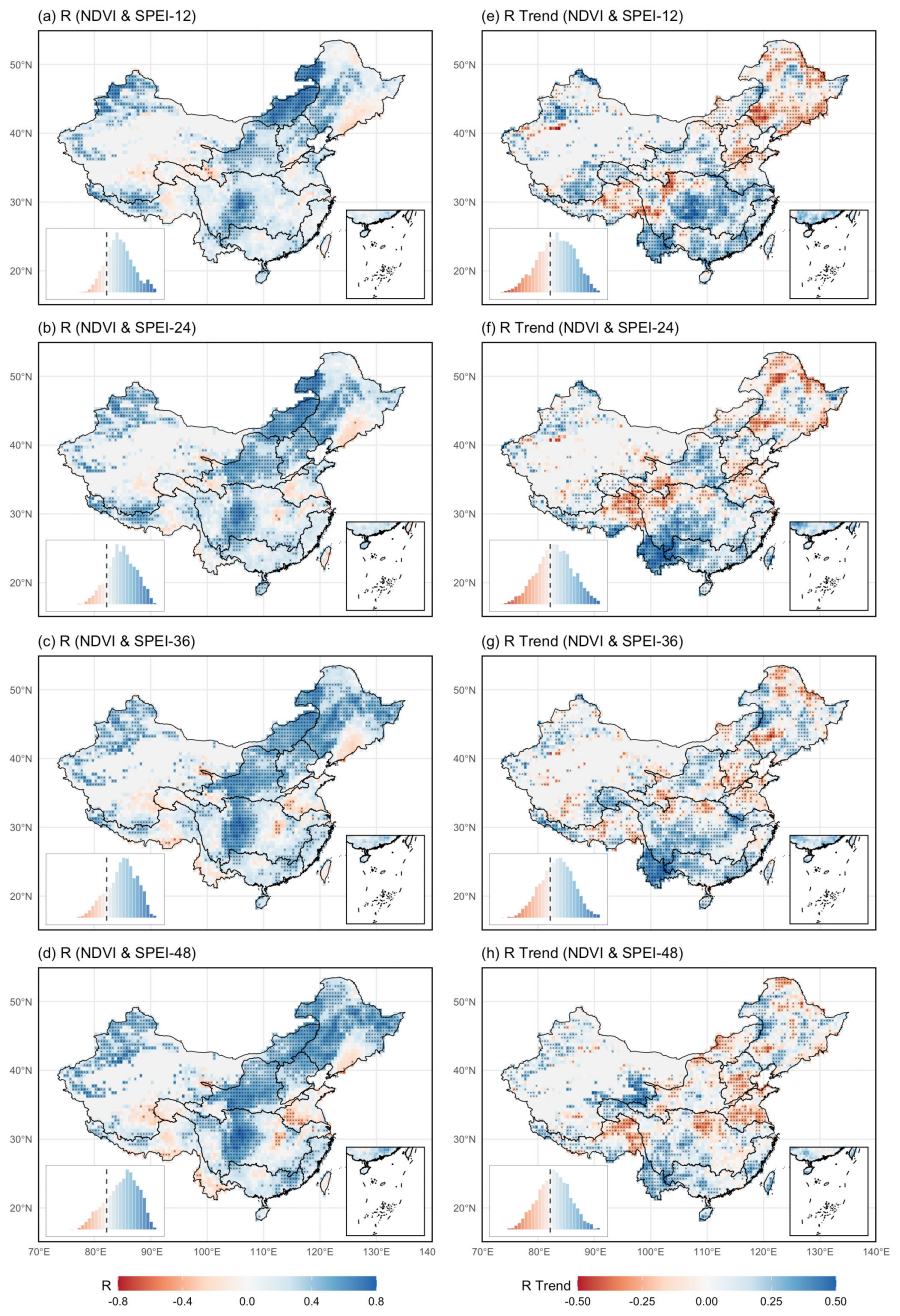
Sensitivity of NDVI to the multiscalar SPEI and its trend across China. **(a–d)** represent the Pearson correlation coefficient (R) between SPEI and NDVI anomalies during 1982–2022. **(e–h)** show the decadal trend of drought sensitivity (R) detected by a 9-year moving window. The inset frequency histograms in the lower-left corner display the distribution of values across all grid cells, with their color fills matching the color-mapping scheme of the corresponding color bar. Grids labeled with black dots indicate that the trend is statistically significant (M-K test: *p* < 0.05).

The relationship between the inter-annual variability of NDVI and multiscalar SPEI exhibited a similar spatial pattern, where positive significant correlation coefficients predominated in the northern basins in China (**Figures 6a–d**). In addition, NDVI across the Sichuan Basin, located in the middle and upper reaches of the Yangtze River Basin, was also remarkably and positively correlated with SPEI (**Figures 6a–d**). Overall, a significant relationship between the inter-annual variability of NDVI and SPEI with different durations was found in 29.09%, 36.60%, 41.36%, and 44.37% of the vegetated lands for SPEI-12, SPEI-24, SPEI-36, and SPEI-48, respectively (**Figures 6a–d**), suggesting that drought is a non-negligible constraint to a greening China. The area with a significantly negative relationship between the inter-annual variability of NDVI and SPEI accounted for less than 2% of the total area in the past four decades (**Figures 6a–d**).

Using a 9-year moving window, we examined the variations of R in each subperiod during 1982–2022. A notably enhanced R (*p* < 0.05), suggesting an increasing sensitivity or risk of surface greenness to drought, was found in vast areas of China (**Figures 6e–h**). The specific proportions, showing a markedly enhanced NDVI sensitivity to SPEI-12, SPEI-24, SPEI-36, and SPEI-48, were 39.67%, 33.04%, 36.21%, and 27.48%, respectively. Most of these lands were located in the Yellow River Basin, the Yangtze River Basin, the lower reaches of the Southwest Basin, and the Pearl River Basin (**Figures 6e–h**). In contrast, a markedly decreasing trend in greenness drought sensitivity, denoting releasing stress from dryness, accounted for 17.81% (SPEI-12), 16.13% (SPEI-24), 12.17% (SPEI-36), and 14.61% (SPEI-48) of the vegetated lands, respectively (**Figures 6e–h**). Interestingly, these areas were mainly distributed in the agricultural regions in the Songhua and Liaohe River Basin, the Haihe River Basin, and the Huaihe River Basin. In addition, the source region of the Yangtze and Yellow Rivers also showed a significantly decreasing R (**Figures 6e–h**).

### 3.4 Sensitivity of greenness to SPEI and its changes at regional scale

#### 3.4.1 The sensitivity across basins, agricultural regions, and plant functional types

**Figure 7:**
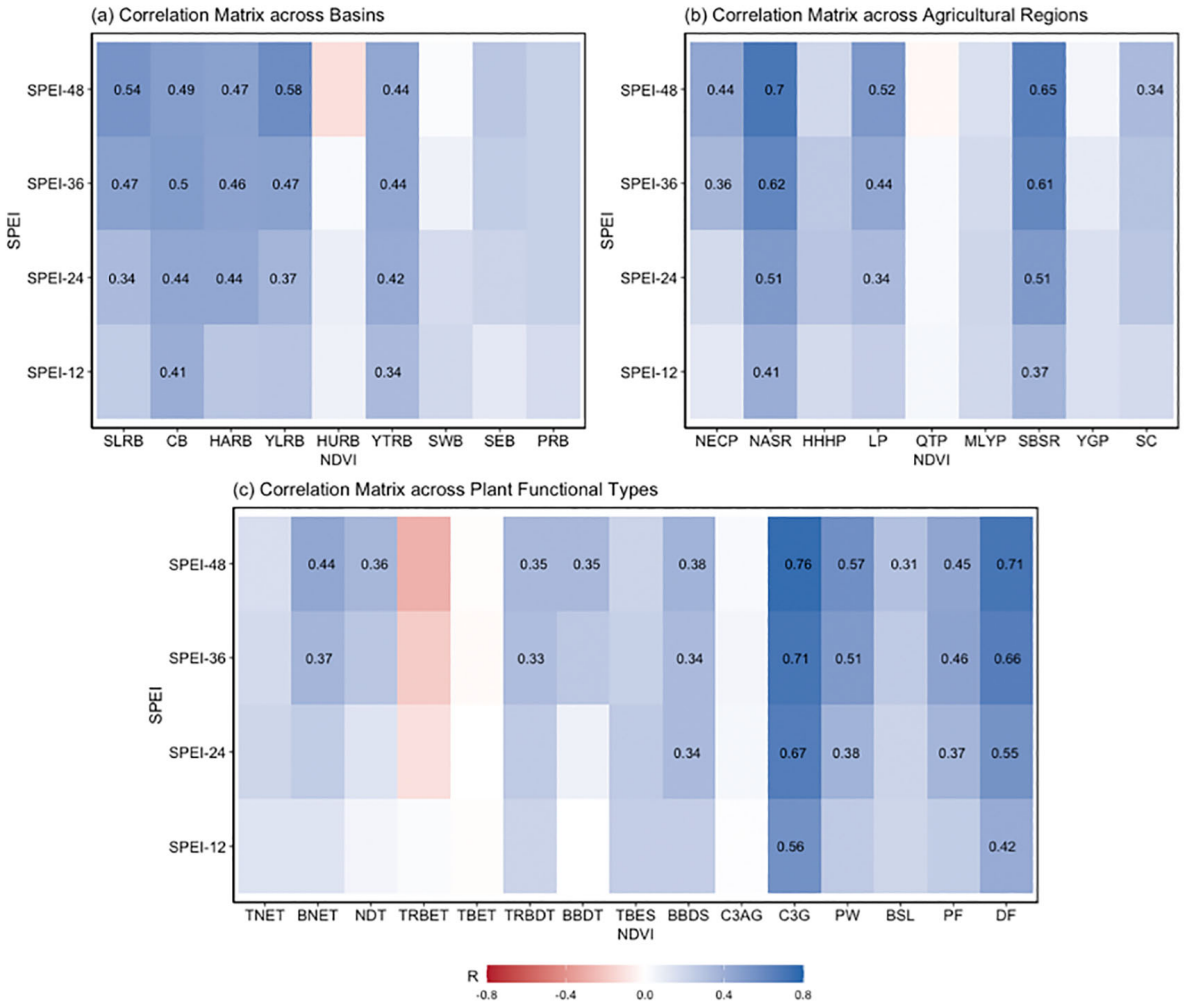
Sensitivity of the interannual variability of NDVI to multi-time scale SPEI across different basins **(a)**, agricultural regions **(b)**, and plant functional types **(c)** in China. SLRB, Song & Liao River Basin, CB, Continental Basin, HARB, Haihe River Basin, YLRB, Yellow River Basin, HURB, Huaihe River Basin, YTRB, Yangtze River Basin, SWB, Southwest Basin, SEB, Southeast Basin, PRB, Pearl River Basin. NECP, Northeast China Plain, NASR, Northern Arid-Semiarid, HHHP, Huang-Huai-Hai Plain, LP, Loess Plateau, QTP, Qinghai Tibet Plateau, MLYP, Middle-lower Yangtze Plain, SBR, Sichuan Basin Regions, YGP, Yunnan-Guizhou Plateau, SC, Southern China. TNET, Temperate Needleleaf Evergreen Tree, BNET, Boreal Needleleaf evergreen Tree, NDT, Needleleaf Deciduous Tree, TRBET, Tropical Broadleaf Evergreen Tree, TBET, Temperate Broadleaf Evergreen Tree, TRBDT, Tropical Broadleaf Deciduous Tree, BBDT, Boreal Broadleaf Deciduous Tree, TBES, Temperate Broadleaf Evergreen Shrub, BBDS, Boreal Broadleaf Deciduous Shrub, C3AG, C3 Arctic Grass, C3G, C3 Grass, PF, Paddy Field, DF, Dry Filed, PW, Permanent Wetlands, UBL, Urban and Built-up Lands, BSL, Barren or Sparsely Lands. R denotes the Pearson correlation coefficient between NDVI variations and SPEI. The inclusion values shown in the matrix plots **(a–c)** indicate that the correlation is statistically significant (*p* < 0.05).

From 1982 to 2022, the NDVI variations in the Huaihe River Basin, the Southwest Basin, the Southeast Basin, and the Pearl River Basin were insensitive to drought within four-year (48-month) durations (**Figure 7a**). The interannual variability of NDVI in the Continental Basin and the Yangtze River Basin was sensitive to the severity of dryness with all the four time-scales (**Figure 7a**). For other basins, including the Haihe River Basin, the Songhua and Liaohe River Basin, and the Yellow River Basin, NDVI variations were significantly correlated with SPEI-24, SPEI-36, and SPEI-48 (**Figure 7a**).

Among different agricultural regions in China, both the Northern Arid-Semiarid Region and the Sichuan Basin-Surrounding Regions showed a significant (*p* < 0.05) relationship between annual NDVI variations and multi-timescale SPEI (**Figure 7b**). Vegetation greenness in the Loess Plateau displayed a notably (*p* < 0.05) correlation, with aridity quantified by SPEI at timescales beyond 12 months (**Figure 7b**). For the Northeast China Plain, NDVI was sensitive to SPEI-36 and SPEI-48, suggesting that vegetation greening was threatened by long-term droughts in these regions (**Figure 7b**). The NDVI variations in the Huang-Huai-Hai Plain (also termed the North China Plain), Middle-lower Yangtze Plain, and the Tibetan Plateau, were insensitive to all four drought agents (**Figure 7b**).

The response of NDVI to SPEI also varied across different plant functional types. Specifically, interannual NDVI variations of the C3 Grass and Dry Field showed a significantly (*p* < 0.05) positive correlation with both annual (SPEI-12) and multi-year (SPEI-24, SPEI-36, SPEI-48) drought conditions (**Figure 7c**). Changes in humidity evaluated beyond 12-month timescales were associated with the variability of NDVI in the Boreal Broadleaf Deciduous Shrub, Permanent Wetlands, and Paddy Field, where positive correlations were found between NDVI and SPEI with multi-year durations (**Figure 7c**). The duration of significant drought stress extended to three years for Boreal Needleleaf Evergreen Trees and Tropical Broadleaf Deciduous Trees, but to four years for Needleleaf Deciduous Trees and Boreal Broadleaf Deciduous Trees, suggesting that long-term droughts are expected to stress vegetation growth (**Figure 7c**).

#### 3.4.2 The trend of greenness drought sensitivity across basins, agricultural regions, and plant functional types

**Figure 8:**
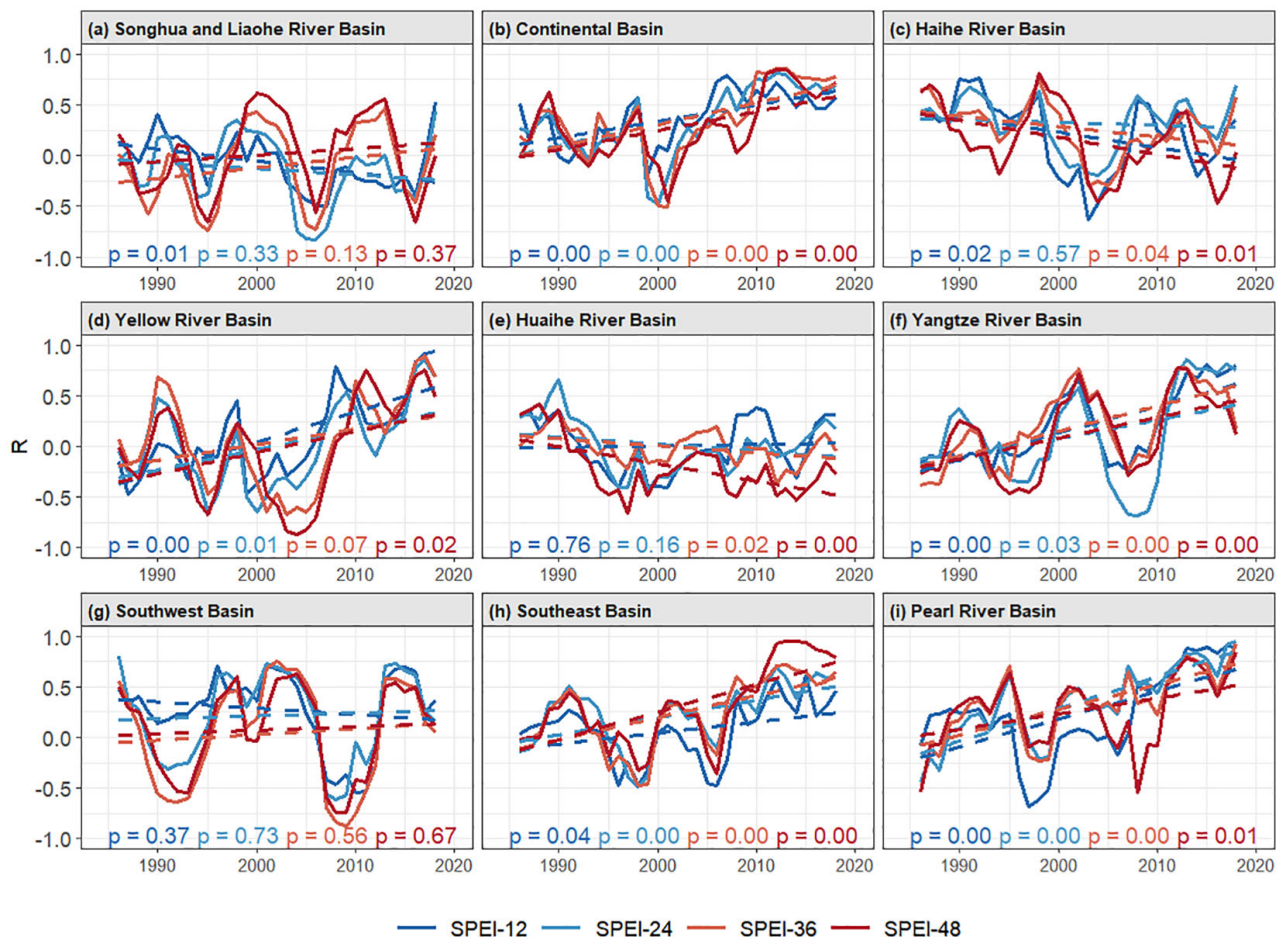
Changes in drought sensitivity across different basins in China. **(a–i)** represent the Song & Liao River Basin, the Continental Basin, the Haihe River Basin, the Yellow River Basin, the Huaihe River Basin, the Yangtze River Basin, the Southwest Basin, Southeast Basin, and the Pearl River Basin, respectively. The blue, light blue, pink, and purple lines represent linear trends of the sensitivity of NDVI anomalies to SPEI-12, SPEI-24, SPEI-36, and SPEI-48, respectively. The p-values with the corresponding color show the significance level of the linear trend detected by the t-test.

The temporal trends of decadal R across the nine basins exhibited distinct spatial heterogeneity. No consistently significant (*p* > 0.05) sensitivity trends were found in the Songhua-Liaohe River Basin and the Southwest Basin (**Figures 8a, g**). Five basins, including the Continental Basin, the Yellow River Basin, the Yangtze River Basin, the Southeast Basin, and the Pearl River Basin, exhibited a substantial increase in the sensitivity of NDVI variations to the four SPEI timescales (**Figures 8b, d, f, h, i**), highlighting the growing impact of drought stress on vegetation dynamics. In the Haihe River Basin and the Huaihe River Basin, the sensitivity displayed a consistent declining trend, particularly for long-term drought conditions (SPEI-36 and SPEI-48) (**Figures 8c, e**), suggesting that the negative effects of drought on vegetation growth have been weakening over time.

**Figure 9:**
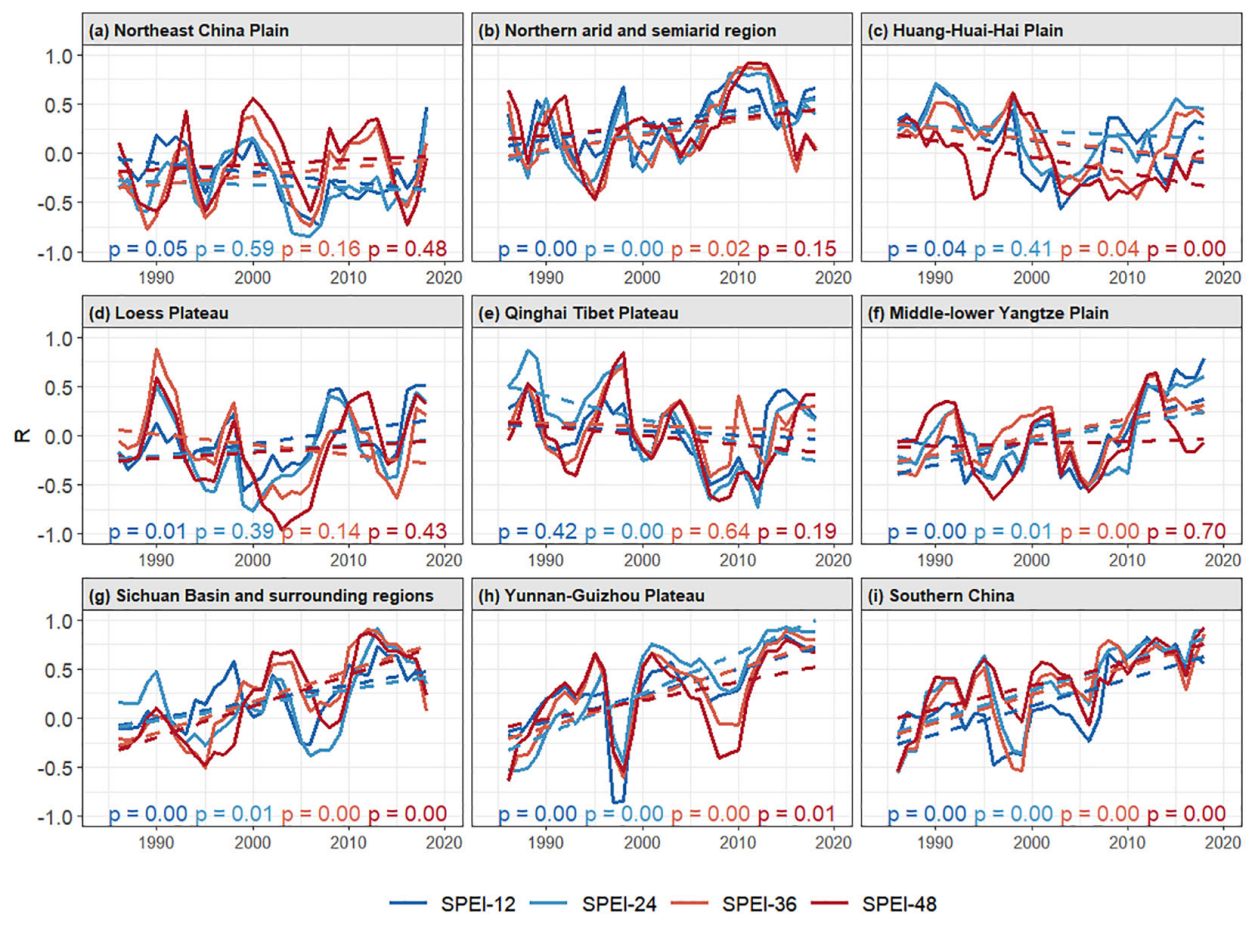
Changes in drought sensitivity across the nine agricultural regions in China. **(a–i)** denote the Northeast China Plain, the Northern Arid-Semiarid, the Huang-Huai-Hai Plain, the Loess Plateau, the Qinghai Tibet Plateau, the Middle-lower Yangtze Plain, the Sichuan Basin Regions, the Yunnan-Guizhou Plateau, and the Southern China, respectively. The blue, light blue, pink, and purple lines represent linear trends of the sensitivity of NDVI anomalies to SPEI-12, SPEI-24, SPEI-36, and SPEI-48, respectively. The p-values with the corresponding color show the significance level of the linear trend detected by the t-test.

A notably positive relationship between NDVI variability and SPEI was detected in the northern arid-semiarid region, the Middle-lower Yangtze Plain, the Sichuan Basin and surrounding regions, the Yunnan-Guizhou Plateau, and Southern China (**Figures 9b, f–g**). The Huang-Huai-Hai Plain, also known as the North China Plain, showed a noticeable decreasing trend in greenness drought sensitivity though it’s not significant for the SPEI-24 (**Figure 9c**). Across lands in the Northeast China Plain, the Loess Plateau, and the Qinghai-Tibet Plateau, no consistently significant (*p* < 0.05) long-term trends were found in the NDVI-SPEI relationships (**Figures 9a, d, e**).

**Figure 10:**
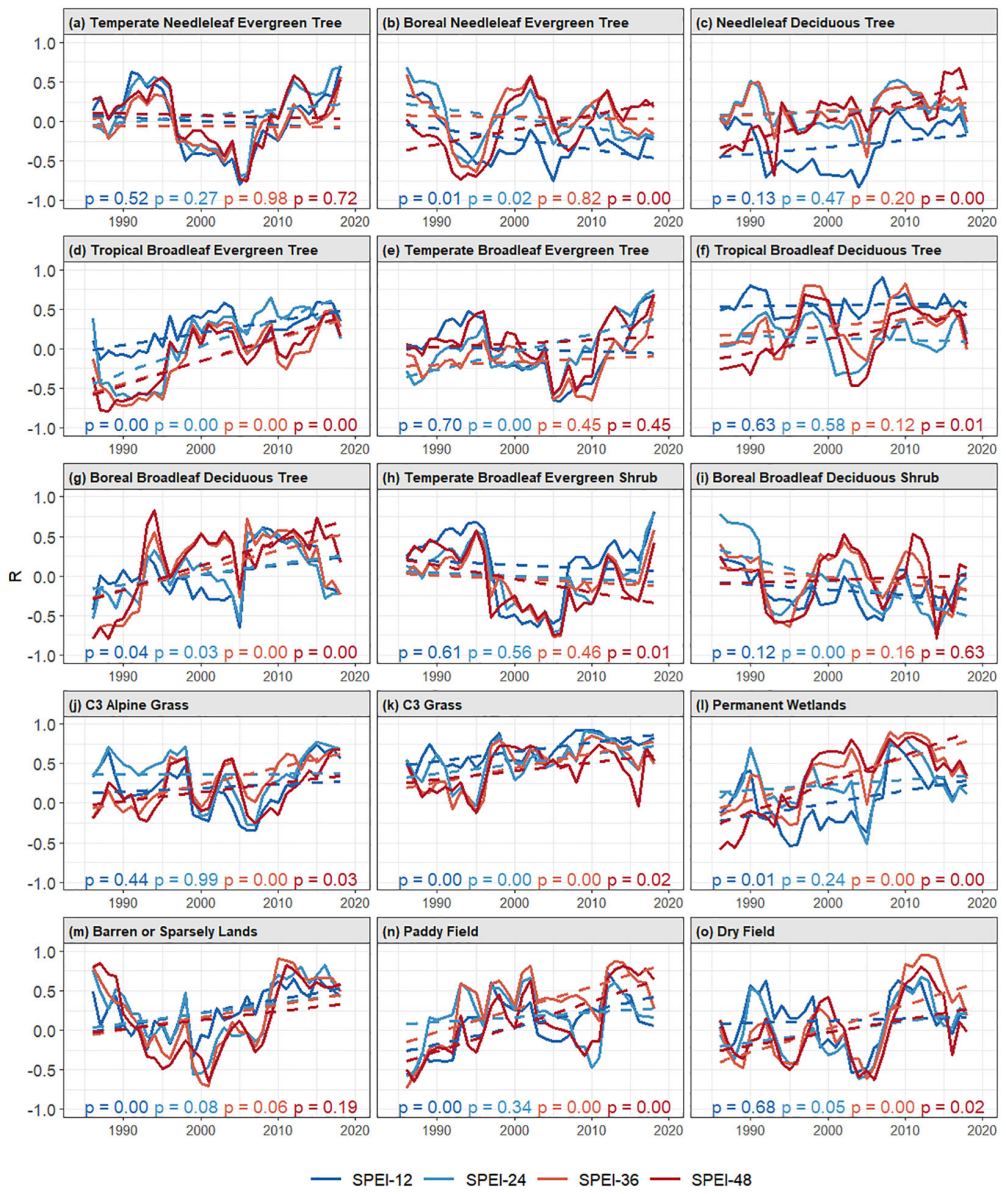
Changes in drought sensitivity across different plant functional types in China. **(a–o)** show the plan functional types of Temperate Needleleaf Evergreen Tree, Boreal Needleleaf evergreen Tree, Needleleaf Deciduous Tree, Tropical Broadleaf Evergreen Tree, Temperate Broadleaf Evergreen Tree, Tropical Broadleaf Deciduous Tree, Boreal Broadleaf Deciduous Tree, Temperate Broadleaf Evergreen Shrub, Boreal Broadleaf Deciduous Shrub, C3 Alpine Grass, C3 Grass, Permanent Wetlands, Barren or Sparsely Lands, Paddy Field, and Dry Filed, respectively. The blue, light blue, pink, and purple lines represent linear trends of the sensitivity of NDVI anomalies to SPEI-12, SPEI-24, SPEI-36, and SPEI-48, respectively. The p-values with the corresponding color show the significance level of the linear trend detected by the t-test.

Three temporal change patterns were identified based on the decadal variations of greenness drought sensitivity. The Temperate Evergreen Trees and Shrubs in China showed a decline in the NDVI drought sensitivity before approximately 2005, followed by a notable increase after 2005 (**Figures 10a, e, h**). The breakpoint year was approximately 2000 for the Barren lands, when the drought risk had substantially enhanced (**Figure 10m**). The Tropical Broadleaf Evergreen Tree, the Boreal Broadleaf Deciduous Tree, the C3 Grass, the Permanent Wetlands, and the Fields (both the Paddy Field and Dry Field) showed an overall consistent increase in the NDVI drought sensitivity (**Figures 10d, g, k, l, n, o**). For the Boreal Needleleaf Evergreen Tree, the Needleleaf Deciduous Tree, the Broadleaf Deciduous Tree, and C3 Alpine Grass, the long-term drought (SPEI-48) sensitivity increased markedly (*p* < 0.05), whereas the yearly and two-yearly aridity sensitivity trend was indistinct or even negative (**Figures 10b, c, f, j**).

## 4 Discussion

### 4.1 Spatiotemporal pattern of vegetation drought sensitivity

It has been widely reported that global warming increases atmospheric water deficits, thereby accelerating surface evapotranspiration and further intensifying regional water scarcity (Condon et al., 2020; Lian et al., 2021). The enhanced atmospheric aridity under a warming climate may exacerbate water consumption, threatening the trade-off between water and carbon sequestration for terrestrial ecosystems (Jiao et al., 2021; Zeng et al., 2023). The global drylands have experienced and will suffer more severe dryness due to water scarcity. Previously, it has been projected by climate models that the drylands will expand rapidly and potentially exacerbate the risk of land desertification and degradation in the near future (Huang et al., 2015; Lian et al., 2021). In this study, we suggest that China has experienced a remarkable increase in dryness since the 1980s (**Figures 3**, **4**). In the northern and northwestern arid and semi-arid regions, especially for the Yellow River Basin and Haihe Basin, a substantial increase in spring dryness has the greatest extent (38.29% of China) and primarily contributes to the notable increase in the multiscalar SPEI (**Figures 2**, **4**). Moreover, the area with a significant wetting trend has been very limited since the 1980s.

The definition and indicator of drought may vary depending on the core elements of concern. Generally, four definitions have been commonly used to characterize drought based on different physical systems, namely meteorological drought, agricultural drought, hydrologic drought, and economic drought (Changnon, 1987; Mishra and Singh, 2010). Generally, meteorological drought is the premonitory of other types of droughts, which propagates through hydrological cycle within multiple timescales and subsequently leads to the presence of other types of droughts (Han et al., 2023; Li et al., 2022). Some studies refer to the drought signal from abnormal meteorological conditions to the variations of agricultural or hydrological processes as “drought propagation” (Crausbay et al., 2017; Eltahir and Yeh, 1999; Li et al., 2022). The propagation time (response time) from meteorological drought to another type of drought can differ for different basins, agricultural regions, and socioeconomic systems (López-Moreno et al., 2013; Ma et al., 2021; Zhang et al., 2022a). There are numerous factors that may regulate drought propagation rate from one to another type, including climatic factors (type, variability, and teleconnection), catchment characteristics (e.g., topography, land cover type, and water storage), and human activities (e.g., irrigation, reservoir operation, water diversion, and groundwater extraction) (Zhang et al., 2022a).

In this study, to analyze the relationship between vegetation growth and drought, we used annual and multi-year SPEI, an indicator of meteorological drought, which can override the “lag effect” of drought and eliminate the confounding effect of drought propagation time (**Supplementary Figures S1**, **S2**). A significant relationship between the inter-annual variability of NDVI and SPEI with different timescales (SPEI-12, SPEI-24, SPEI-36, and SPEI-48) was found across 29.09%, 36.60%, 41.36%, and 44.37% of the vegetated lands in China, respectively (**Figures 6a–d**), suggesting that more NDVI variations can be explained by the accumulate water deficit effect beyond this year. At the basin scale, agricultural regions, and plant functional scale, the relationship between NDVI variability and SPEI is consistent with our understanding. For example, the interannual NDVI variations in the northern basins of China, including the Songhua and Liaohe River Basin, Continental Basin, Haihe River Basin, and Yellow River Basin, were significantly correlated with SPEI (**Figure 7a**). For the southern basins including the Huaihe River Basin, Southwest Basin, Southeast Basin and Pearl River Basin, where the water resources are relatively abundant, the relationships between NDVI variability and SPEI are insignificant across all the selected time scales (**Figure 7a**). At the Yangtze River basin scale, NDVI variations were sensitive to SPEI changes (**Figure 7a**), which was mainly attributed to the significant relationship across the Sichuan Basin and its surrounding areas (**Figures 6a–d**). Besides, it is worth noting that the relationship between SPEI and NDVI variations at the 12-month scale was unclear for most basins, agricultural regions, and plant functional types (**Figure 7**). In contrast, dryness beyond the annual timescale, namely SPEI-24, SPEI-36 and SPEI-48, significantly affected NDVI variability. Therefore, the “lag effect” of meteorological drought must be fully considered, and the appropriate time scale should be determined to fully capture the drought consequences.

### 4.2 Escalating drought sensitivity of vegetation and its potential drivers

Previously, it is widely reported that the lands with enhanced water constraints on vegetation growth have significantly expanded on the terrestrial surface under warming (Denissen et al., 2022; Jiao et al., 2021; Wang et al., 2024; Wei et al., 2023; Zhang et al., 2022b). In this study, we suggest that nearly 55% of the vegetated lands in China show a greening trend in the past four decades (**Figure 5**). Meanwhile, the sensitivity of NDVI variations to SPEI also increased remarkably, and the sensitivity of NDVI variations to SPEI-12, SPEI-24, SPEI-36 and SPEI-48 show significant increasing trend across 39.67%, 33.04%, 36.21%, and 27.48% of vegetated lands in China, respectively (**Figures 6e–h**). The escalating greenness drought sensitivity was also evident in basins, agricultural regions, and plant functional types (**Figures 8**–**10**).

Several drivers, including changes in water availability, drought regimes, biological adjustments, and ecosystem management practices, potentially regulate the sensitivity of greenness to drought. Declining water availability, often driven by reduced precipitation and enhanced ET, can amplify ecosystem vulnerability to drought. At the global scale, Chen et al. (2022b) revealed that vegetation greening has prominently exacerbated the decline in water availability (precipitation minus evapotranspiration) by enhancing ET in drylands. Cui et al. (2022) also suggested that enhanced vegetation growth has been associated with increased evapotranspiration (ET), which may lead to regional water deficits. Recently, Chen et al. (2025) demonstrated that the accelerated greening of vegetation has enhanced the shrinkage of regional terrestrial water storage (TWS) in the Loess Plateau (not the agricultural Loess Plateau referred to in this study) since 2000, when the revegetation project was implemented. This may potentially enhance the drought sensitivity of vegetation and drought-induced carbon sequestration loss. In this study, we indeed found that the SPEI sensitivity of NDVI has enhanced across the Loess Plateau, the main body of the Yellow River Basin (**Figures 6**, **7d**). Meanwhile, we also find that the drought sensitivity of drylands in the Continental Basin shows a significantly positive trend (**Figures 6**, **7b**).

Alterations in drought regimes, including frequency and intensity, may also regulate the drought sensitivity of vegetation growth. The increasing frequency and magnitude of extreme droughts may exceed the adaptive capacity of vegetation, particularly under compound events involving both drought and heat stress. In the study conducted by Wu et al. (2019), they concluded that the frequency of compound dry/warm extremes showed a significant increasing trend across most of China from 1961 to 2014. These compound extremes have been shown to significantly suppress vegetation growth rates and productivity (Anderegg et al., 2012; Liu et al., 2023; Lu et al., 2023), which is reflected in the increasing sensitivity of vegetation growth to drought. In addition, we find that the proportion of areas with a significant increasing trend in the sensitivity of NDVI to SPEI at the inter-annual scale (SPEI-12) is 39.67%, which is higher than the percentages at 2–4 years scales (**Figures 6e–h**). The dominant increase in short-term drought sensitivity observed in some regions may reflect the rising occurrence of short-term drought events and heat stress (e.g., flash drought and heat waves) with abrupt onset and intensifying severity, which has been described in previous global studies (Christian et al., 2021; Piao et al., 2014; Wei et al., 2023).

The biological responses of vegetation to drought, relating to adjustments in plant traits and community species composition, are important biological factors that may affect water use efficiency and the sensitivity of photosynthetic productivity to drought. For instance, woody plants can adjust above- and below-ground physiological traits in response to sustained drought (Rowland et al., 2023; Vadez et al., 2024). Community structure can shift toward a greater abundance of drought-tolerant, deep-rooted species, which enhances water acquisition, increases drought resilience, and helps maintain stable ecosystem productivity (Batllori et al., 2020; Liu et al., 2018). Other environmental factors may also play a role, such as increasing atmospheric CO_2_ and nitrogen availability. These factors can directly affect plant traits and water use efficiency or indirectly compensate for photosynthetic loss caused by drought (Jiao et al., 2021; Kicklighter et al., 2019; Li et al., 2023a; Zhang et al., 2022b). Consequently, these processes collectively influence the sensitivity of vegetation to drought stress.

Ecosystem management practices also play a crucial role in enhancing and alleviating the adverse consequences of drought. For croplands, anthropogenic water extraction for irrigation can significantly reduce drought stress on grain yield, resulting in the insensitivity of farmland vegetation to drought (Zhao et al., 2023). In the agricultural regions across China, we found the sensitivity of NDVI to SPEI had no noticeable change for the Northeast China Plain, the Loess Plateau, and the Qinghai Tibet Plateau (**Figures 9a, d, e**). Specifically, the NDVI drought sensitivity subsequently decreased across the Huang-Huai-Hai Plain (also known as the North China Plain) (**Figures 6**, **9c**), where the groundwater has been overexploited as a result of irrigation water demand (Yang et al., 2022). Other crop management practices like fertilization can also alleviate the negative impacts of drought and high temperature (Li et al., 2023a; Zhao et al., 2023). Besides, intensive human-induced greenness change, such as afforestation/deforestation and land cover change, may decouple the relationship between vegetation growth and drought, resulting in an obvious decrease in the statistical correlation between vegetation indices and drought indicators. On the other hand, ecosystem management strategies can also enhance the sensitivity of ecosystem functions to drought. In grasslands, for instance, reduced grazing pressure may enhance the linkage between vegetation growth and climatic variability. Some studies suggest that this can be used as a criterion for judging the intensity of human activities (Chen et al., 2014). Therefore, it should be clarified that the escalating drought sensitivity of vegetation may also be attributed to the decoupling between vegetation and human-induced stress in China, where many ecological restoration projects have been implemented and contributed remarkably to a greening Earth (Chen et al., 2019; Feng et al., 2016; Li et al., 2021; Wang et al., 2015). Nevertheless, the impacts and mechanisms of drought on the sustainability of vegetation greening are still vital questions worthy of further study.

### 4.3 Uncertainties and limitations

We explicitly acknowledge that optical satellite observations, specifically the NDVI, are susceptible to historical sensor constraints and asymptotic canopy saturation over dense vegetation zones. The primary justification for selecting the four-decade NDVI as our baseline data stream is its extensive temporal coverage, which is critical for preserving a sufficiently long R time series. Because a 9-year moving window mathematically contracts the diagnostic timeline by 8 years, a long historical archive is indispensable for establishing reliable long-term evolutionary trends. To evaluate the potential uncertainties introduced by data source and the saturation effect, we cross-validated our baseline calculations (**Figure 6**) against three saturation-resistant metrics: Enhanced Vegetation Index (EVI), Solar-Induced Chlorophyll Fluorescence (SIF), and Vegetation Optical Depth (VOD) (**Supplementary Figure S3**). Crucially, these three vegetation proxies are derived from distinct sensor mechanisms, ranging from advanced optical reflectance and active physiological photochemistry to passive microwave remote sensing, and their robust capabilities in mitigating or entirely bypassing canopy saturation effects have been widely recognized and validated across the remote sensing community. Although these proxies represent structurally diverse indicators, their resulting sensitivity trends consistently reproduced a macro-scale intensification of the vegetation-drought coupling across China. While their shorter temporal baselines (primarily post-2000) compromise the statistical degrees of freedom required for robust long-term moving-window regressions, their directional alignment with our 40-year NDVI trajectories confirms that localized saturation artifacts do not undermine the scientific validity or the macro-scale conclusions of this study.

Furthermore, the configuration of the temporal window length introduces mathematical uncertainty in our moving-window correlation analysis. To evaluate the statistical robustness of the 9-year sliding window utilized in our baseline framework, we performed a pixel-level sensitivity test by systematically re-calculating the decadal trends of the NDVI–SPEI coupling (R trend) under alternative window configurations of 5 years and 15 years (**Supplementary Figure S4**). Our spatial and statistical cross-validation revealed that the 5-year moving window yielded patterns with pronounced numerical instability and noise, displaying substantial divergence from the 9-year baseline results. This discrepancy is primarily attributed to small-sample artifacts, where short integration periods amplify the leverage of single-year anomalies and overly penalize significance tests. Conversely, the 15-year and 9-year window configurations exhibited an exceptional degree of spatial congruence and trend magnitude consistency. Nevertheless, an extended window inherently contracts the operational timeline of the resulting R time series (e.g., a 15-year window truncates the sequence by 14 years). Therefore, to strike an optimal trade-off between maximizing statistical degrees of freedom for robust regression testing and preserving sufficient temporal resolution to capture sub-decadal variations, the 9-year moving window was retained as a scientifically sound configuration for this multi-decadal study.

## 5 Conclusion

Drought plays a critical role in threatening China’s greening, as greenness dynamics across extensive vegetated lands are significantly influenced by drought indicators. Notably, the sensitivity of vegetation greenness to drought has substantially enhanced across nearly 40% of vegetated areas, suggesting that drought is becoming an escalating constraint to a greener China, albeit a remarkable greening trend widely observed in nearly 55% of lands since the 1980s. Specifically, five of nine drainage basins (the Yellow River Basin, the Yangtze River Basin, the Continental Basin, the Southeast Basin, and the Pearl River Basin), four of nine agricultural regions (the Middle-lower Yangtze Plain, the Sichuan Basin, the Northern Arid-Semiarid, and the Yunnan-Guizhou Plateau), and six of fifteen plant functional types (the Tropical Broadleaf Evergreen Trees, the Boreal Deciduous Trees, the C3 Grasses, the Wetlands, and the Croplands) exhibited substantial increase in drought sensitivity. Both natural and anthropogenic factors potentially contribute to the shifting drought sensitivity of vegetation; however, this study exclusively focuses on a statistical approach to reveal the intensifying empirical coupling between greenness and drought indices, meaning that a more rigorous, quantitative attribution framework remains a critical directive for future research. A comprehensive understanding of these dynamics is essential to assess potential ecological challenges under a changing climate and support a sustainably greening China.

## Data Availability

Publicly available datasets were analyzed in this study. This data can be found here: SPEIbase v2.9.0: https://spei.csic.es/; PKU GIMMS NDVI (Version 1.2) https://essd.copernicus.org/articles/15/4181/2023/essd-15-4181-2023-assets.html.
