# Supplementary material for: Escalating drought sensitivity of a greening China
Source: Front Plant Sci. 2026 Aug 5;17:1873724. doi: 10.3389/fpls.2026.1873724 (PMC13485981; doi:10.3389/fpls.2026.1873724)
Supplement: Supplementary file 1 [file Supplementaryfile1.docx]

Supplementary Material

**Fig. S1** The distribution of correlation (R) between annual mean NDVI and SPEI across scales (1-48 month) by basins in China. SLRB: Song & Liao River Basin, CB: Continental Basin, HARB: Haihe River Basin, YLRB: Yellow River Basin, HURB: Huaihe River Basin, YTRB: Yangtze River Basin, SWB: Southwest Basin, SEB: Southeast Basin, PRB: Pearl River Basin. It should be noted that to remove some outlier values, we kept the pixel values between the 10th and 90th percentiles.

**Fig. S2** The distribution of correlation (R) between annual mean NDVI and SPEI across scales (1-48 month) by agricultural regions. NECP: Northeast China Plain, NASR: Northern Arid-Semiarid, HHHP: Huang-Huai-Hai Plain, LP: Loess Plateau, QTP: Qinghai Tibet Plateau, MLYP: Middle-lower Yangtze Plain, SBR: Sichuan Basin Regions, YGP: Yunnan-Guizhou Plateau, SC: Southern China. It should be noted that to remove some outlier values, we kept the pixel values between the 10th and 90th percentiles.

**Fig. S3** Spatial patterns of R trends across multiple satellite-based proxies and accumulating SPEI scales. EVI: Enhanced Vegetation Index (2000–2022), SIF: Solar-Induced Chlorophyll Fluorescence (2000–2022), and VOD: Vegetation Optical Depth (1987–2021).

**Fig. S4** Decadal R trend across China detected by a 5-year moving window (a), 9-year moving window, and 15-year moving window.
